# Predicting task-general mind-wandering with EEG

**DOI:** 10.3758/s13415-019-00707-1

**Published:** 2019-03-08

**Authors:** Christina Yi Jin, Jelmer P. Borst, Marieke K. van Vugt

**Affiliations:** Bernoulli Institute for Mathematics, Computer Science and Artificial Intelligence, University of Groningen, Nijenborgh 9, 9747AG Groningen, Netherlands

**Keywords:** Mind-wandering, Spontaneous thought, Single-trial ERP, EEG, Support vector machine, Sustained attention to response task, Alpha oscillations

## Abstract

Mind-wandering refers to the process of thinking task-unrelated thoughts while performing a task. The dynamics of mind-wandering remain elusive because it is difficult to track when someone’s mind is wandering based only on behavior. The goal of this study is to develop a machine-learning classifier that can determine someone’s mind-wandering state online using electroencephalography (EEG) in a way that generalizes across tasks. In particular, we trained machine-learning models on EEG markers to classify the participants’ current state as either mind-wandering or on-task. To be able to examine the task generality of the classifier, two different paradigms were adopted in this study: a sustained attention to response task (SART) and a visual search task. In both tasks, probe questions asking for a self-report of the thoughts at that moment were inserted at random moments, and participants’ responses to the probes were used to create labels for the classifier. The 6 trials preceding an off-task response were labeled as mind-wandering, whereas the 6 trials predicting an on-task response were labeled as on-task. The EEG markers used as features for the classifier included single-trial P1, N1, and P3, the power and coherence in the theta (4–8 Hz) and alpha (8.5–12 Hz) bands at PO7, Pz, PO8, and Fz. We used a support vector machine as the training algorithm to learn the connection between EEG markers and the current mind-wandering state. We were able to distinguish between on-task and off-task thinking with an accuracy ranging from 0.50 to 0.85. Moreover, the classifiers were task-general: The average accuracy in across-task prediction was 60%, which was above chance level. Among all the extracted EEG markers, alpha power was most predictive of mind-wandering.

**Electronic supplementary material:**

The online version of this article (10.3758/s13415-019-00707-1) contains supplementary material, which is available to authorized users.

Mind-wandering—also referred to as task-unrelated thinking—is a common phenomenon. It is associated with both advantages and problems in our daily life. While disrupting performance of the ongoing task, mind-wandering could also help with future planning and problem solving (Smallwood & Schooler, 2015). Throughout the literature, researchers have studied this mental phenomenon through different perspectives and defined it in different ways. The most straightforward definition of mind-wandering might be “off-task thought” or “task-unrelated thought” (Barron, Riby, Greer, & Smallwood, 2011; McVay & Kane, 2009). Here, mind-wandering is defined by its content—which is irrelevant to the ongoing task. This definition mostly fits our daily experience. However, the task-unrelated thought definition is perhaps too general, in that it also contains thoughts that are triggered by environmental distractors such as sounds or smells. Some researchers do not consider such stimulus-driven thoughts as mind-wandering and restrict mind-wandering to “stimulus-independent thought” (Smallwood & Schooler, 2015). They focus on the importance of the generation of these thoughts, which they argue should be self-generated (Schooler et al., 2011; Smallwood & Schooler, 2015). Other researchers believe that what is crucial about mind-wandering is that it is spontaneous and not constrained by anything (Christoff, Irving, Fox, Spreng, & Andrews-Hanna, 2016). For the purposes of the current study, we define mind wandering as self-generated, task-unrelated thought.

Several explanations of self-generated, task-unrelated thought have been put forward. The prominent *executive attentional framework* of mind-wandering predicts that mental effort devoted to the primary task is reduced because mind-wandering processes consume part of the cognitive resources (Smallwood & Schooler, 2006, 2015). Indeed, in event-related potential (ERP) studies, an electrophysiological index of cognitive processing—the P3—was shown to be reduced when participants engaged in mind-wandering processes, compared with when they were in an on-task state (Smallwood, Beach, Schooler, & Handy, 2008). Furthermore, functional magnetic resonance imaging (fMRI) studies have shown that mind-wandering is associated with the involvement of the executive network regions (Christoff, Gordon, Smallwood, Smith, & Schooler, 2009). However, although the frontoparietal network was active, activation during mind-wandering was less than during the on-task state (Christoff et al., 2016; Kirschner, Kam, Handy, & Ward, 2012; Mittner et al., 2014).

The relation between mind-wandering and executive functions is more complex, however. For instance, working memory capacity plays a role in resisting mind-wandering (Robison & Unsworth, 2015). Furthermore, while for people with low working memory capacity mind-wandering tends to occur independently of the context, people with higher working memory capacity tend to mind-wander more strategically (Robison & Unsworth, 2017). In a similar vein, mind-wandering has been divided into intentional and unintentional mind-wandering, which have different neural correlates and functional consequences. Some studies have found that intentional mind-wandering occurs more often in easy task conditions compared with difficult conditions. On the contrary, unintentional mind-wandering occurs more often in difficult conditions than in easy ones (Seli, Risko, & Smilek, 2016).

An interesting finding is that mind-wandering not only consumes cognitive resources but also inhibits the perceptual processing of the external stimuli, a phenomenon that has been referred to as “perceptual decoupling” (Schooler et al., 2011). Mind-wandering was shown to be accompanied by reduced P1 and N1 (Kam et al., 2011). Since both P1 and N1 are very early ERP components indexing processing during the sensory input stage, their reduction is taken as evidence supporting an inhibitory effect of mind-wandering on external perception. This inhibitory effect might be a possible way of protecting the internal train of thought against getting interrupted (Kam & Handy, 2013), but this is still speculative. Following this idea, mind-wandering has also been referred to as “decoupled thought,” as in, decoupled from the environment.

Mind-wandering is typically studied experimentally by using the experience-sampling methodology. In experience-sampling experiments, probe questions are randomly inserted in the task of interest, asking for subjects’ self-report about their thoughts and feelings, which is then used to mark discrete time points as mind-wandering. Such self-reports are necessary, because mind-wandering occurs automatically and implicitly by definition, so that researchers cannot control it through experimental manipulations. Instead, they can only detect it. The thought probe methodology has obvious drawbacks as well: (1) probes interrupt the ongoing train of thought, causing unwanted interference, and (2) because of this interference, probes cannot be used too often, with the result that continuous detection of mind-wandering can hardly be realized. In other words, the dynamics of mind-wandering cannot be studied merely through experience sampling.

If there were a neurophysiological measure to differentiate between mind-wandering and on-task behavior without the need for interruptions, the problems mentioned above would disappear. While there is promising work along these lines using a combination of fMRI and eye tracking (Mittner et al., 2014), no mind-wandering classifiers have been built for EEG. This leads us to the primary goal of this study: to train a machine-learning classifier that can detect mind-wandering based on electroencephalography (EEG) data. Based on the associated features of mind-wandering—reduced task-relevant cognitive processing and reduced sensory processing—we propose EEG markers reflecting those processes to be candidate features for the classifier. These are the bilateral occipital P1 and N1 as indices of visual perceptual processing, parietal P3 as an indication of manipulations in working memory during task performance, the power of the alpha band (8.5–12 Hz) as an index of sensory processing when being examined at parietal-occipital electrodes (Ergenoglu et al., 2004), the power of the theta band (4–8 Hz) as an indication of task-relevant processing and cognitive control (Cavanagh & Frank, 2014; Harper, Malone, & Iacono, 2017), and the phase coherence between electrodes as an index of the interregional communication during task performance (Cavanagh, Cohen, & Allen, 2009).

Two tasks were employed in the current research to ensure generalizability of the classifier. The first task, the sustained attention to response task (SART), has been used in several mind-wandering studies. Subjects should respond to the frequently appearing nontarget stimulus and withhold their response to the infrequently appearing targets. Mind-wandering is likely to emerge here because the task is easy and boring. Previous studies using the SART demonstrated decreased accuracy when subjects were mind-wandering (e.g., Kam et al., 2011; McVay & Kane, 2013; Smallwood et al., 2008; van Vugt & Broers, 2016). To examine the task dependency of the EEG markers identified, we also included another paradigm in this study that contrasted with the SART in relying more on processing of external stimuli: a visual search task. To classify trials into mind-wandering and on-task states, we used the experience sampling method in both tasks to obtain subjective judgments of the participant’s mental states.

## Method

### Subjects

A total of 30 subjects took part in the experiment. Subjects reported normal or corrected-to-normal vision and were proficient in written and spoken English. After the preprocessing, EEG data of 12 subjects were not entered the further analyses as they had fewer than 30 trials in one of the mental states per task. This was caused by artifacts in the recorded EEG signal in combination with fewer reports in one of the two mental states. The data reported are from the remaining 18 subjects (13 females, ages 18–30 years, *M* = 23.33, *SD* = 2.81 ). One of them was left-handed. For the participants included, at least 72% of the collected trials remained. In the SART, the mean trial count was 97.33 (*SD* = 40.14) in the on-task class and 98.33 (*SD =* 37.50) in mind-wandering. In the visual search task, the mean trial count was 132.90 (*SD* = 42.15) for on-task and 89.61 (*SD* = 44.99) for mind-wandering. The research was conducted following the Declaration of Helsinki. Subjects gave written informed consent. They were paid 40 euros total for participation in two experimental sessions of 2.5 hours each.

### Procedure

The experiment included two sessions, each lasting approximately 2.5 h, including the EEG setup time. Participants performed the experiment in a sound-attenuating booth. The tasks were programmed and presented in OpenSesame (Mathôt, Schreij, & Theeuwes, 2012). EEG was continuously recorded during the tasks with a Biosemi 128-channel system.

**Fig. 1:**
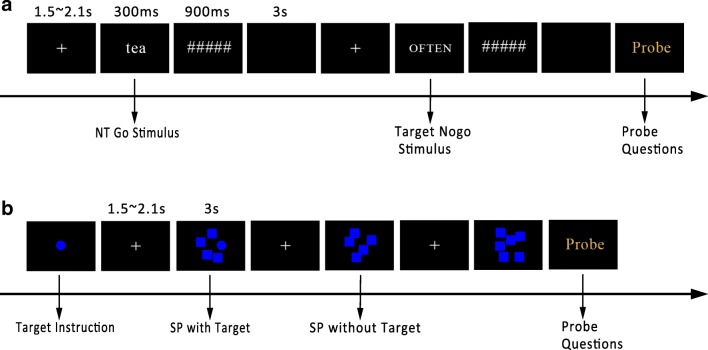
Experimental procedure. **a** In the SART, every trial started with a fixation cross, followed by a word for 300 ms and a mask for 900 ms. There was a 3-s blank as the intertrial interval (ITI). Two types of stimuli are illustrated: a lowercase word (*tea*) as the go stimuli, and an uppercase word (*OFTEN*) as the no-go stimulus, which was the target. Probes always occurred after a no-go trial. **b** In the visual search task, every trial started with a fixation cross, followed by a search panel for 3 s. Two consecutive probes were separated by 7 ~ 24 trials. A visual search target was present on half of the trials and absent on the other half. NT = nontarget; SP = search panel. (Color figure online)

In the SART, white stimuli were presented in the center of the screen against a black background (see Fig. 1a). Each trial began with a fixation cross for a uniformly sampled period between 1.5 s ~ 2.1 s. Each word stimulus appeared for 300 ms followed by a 900-ms mask. The intertrial interval was 3 s. Word stimuli subtended a visual angle of approximately 0.75° vertically and 1.5° ~ 10.5° horizontally. Participants were instructed to press “m” whenever they saw a frequent lowercase word (i.e., nontarget) that occurred 89% of the time and to withhold responding when they saw a rare uppercase word (i.e., target) that occurred 11% of the time.

In the visual search task, blue stimuli were presented in the center of the screen against a black background. At the beginning of each block, there was an instruction about the target to search. Each trial began with a fixation cross for a period of 1.5 s ~ 2.1 s. Each search panel appears for 3 s with a visual angle of 7.47° × 7.47° both horizontally and vertically. Participants were instructed to tell whether a target was presented by pressing the left arrow for “yes” and the right arrow for “no.” There was an equal probability of the target-present and the target-absent trials.

Tasks included 12 blocks (six blocks for each task in each session). A SART block has 135 trials and a visual search block has 140 trials. The timeline of each trial is depicted in Fig. 1.

The subjects were seated at a distance of approximately 60 cm in front of the display. They were instructed to remain still, keep their eyes focused on the screen, and refrain from blinking while performing the tasks. The sequence of task administration was counterbalanced across subjects and sessions. Subjects were given breaks between blocks while the experimenter checked and corrected the electrode impedances.

### Stimuli

The materials for the SART were 283 English words of regular usage, for example, geographical locations (e.g., America), nature (e.g*.,* sea), time (e.g., evening), and other categories (see Appendix A for the full list of words). Word length ranged from two to 14 letters. The words were taken from a previous study of mind-wandering (van Vugt & Broers, 2016).

In the visual search task, materials were square search panels consisting of 4–8 squares or circles of equal size. The target to search could be either a square or circle, and a target was present on half of the trials.

### Experience-sampling thought probes

Both tasks were interrupted by probe questions, asking subjects to report their thoughts at that moment. Subjects could choose one of six options: (1) I entirely concentrated on the ongoing task; (2) I evaluated aspects of the task (e.g., my performance or how long it takes); (3) I thought about personal matters; (4) I was distracted by my surroundings (e.g., noise, temperature, my physical condition); (5) I was daydreaming, thinking of task unrelated things; (6) I was not paying attention, but my thought wasn’t anywhere specifically. These thought probes were derived from our previous experiments (Huijser, van Vugt, & Taatgen, 2018). Participants answered the questions by pressing the corresponding number on the keyboard.

In the SART, probes always appeared after a no-go trial. There were 54 probe questions in each task. Two consecutive probes were separated by 7–24 trials, which meant thought probes occurred roughly every 34–144 seconds.

### EEG recording and offline processing

Continuous EEG was recorded by a Biosemi 128-channel system with six additional electrodes used to detect eye movements and measure mastoid signals. The sampling rate was 512 Hz. An electrode next to the vertex electrode was used as the reference during recording. Impedances were kept below 40 kΩ. Off-line EEG preprocessing was performed with the EEGLAB toolbox (Version13.6.5b; Delorme & Makeig, 2004) in MATLAB (Version 2013b).

For off-line analysis, continuous data were rereferenced to the average signal of mastoids, band-pass filtered (0.5–40 Hz), down-sampled to 256 Hz, and segmented into epochs of 1,600 ms (400 ms before and 1,200 ms after stimulus onset). Bad channels were identified by visual inspection (channels with excessive spikes or with a noisier signal than surrounding channels) and replaced through spherical interpolation before artifact rejection. We performed infomax independent component analysis (ICA) for ocular artifact detection and removal. Additionally, data segments were inspected visually to screen for artifacts.

### Data analysis

#### Trial classification

Six trials proceeding each probe were analyzed, accounting for roughly 30–36 seconds.^1^ This practice followed the assumption that the periodic fluctuations in attention might be supported by very low frequency (0.01–0.1 Hz) coherence within the default mode network (Sonuga-Barke & Castellanos, 2007), and, typically mind-wandering would persist for more than a single trial (Bastian & Sackur, 2013). Trials selected were either labeled as mind-wandering or on-task state based on subjects’ responses to the probes. Probe Responses 1 (I entirely concentrated on the ongoing task) and 2 (I evaluated aspects of the task) were defined as a task-related mental activity. Responses 3 (I thought about personal matters) and 5 (I was daydreaming, thinking of task unrelated things) indicated self-generated task-unrelated thoughts; thus, they were considered as mind-wandering.^2^ There were two particular cases that did not fit either category. Response 4 (I was distracted by my surroundings) indicated off-task thought triggered by the external environment or from body sensations. This kind of thinking is usually classified as distraction instead of mind-wandering (Christoff et al., 2016). In addition, Response 6 (I was not paying attention, but my thought wasn’t anywhere specifically) indicates a mind-blanking state without involvement in self-generated thoughts (Ward & Wegner, 2013). These responses were excluded from further analyses. These thought categories accounted for 1.85%–30.56% of the total reports across subjects (*M* = 16.05%, *SD* = 8.86%).

#### Behavioral measures

We computed accuracy and average response time of the correct trials for each mental state for each task for each subject. Performance in different mental states was compared using paired *t* tests. Effect size was reported as Cohen’s *d*.

#### Single-trial ERP

To detect EEG components in each trial, we used the single-trial ERP methodology (Bostanov, 2004; Bostanov & Kotchoubey, 2006). Different from traditional ERP analysis, which averages the signal across trials for noise removal, single-trial ERP first builds an ideal ERP waveform using a Mexican hat function:

1 $$ \psi (t)=\left(1-16{t}^2\right){e}^{-8{t}^2} $$

and computes its cross-covariance with the single-trial EEG signal

2 $$ W\left(s,t\right)=\frac{1}{\sqrt{s}}\underset{-\infty }{\overset{\infty }{\int }}f\left(\tau \right)\psi \left(\frac{\tau -t}{s}\right) d\tau . $$

**Fig. 2:**
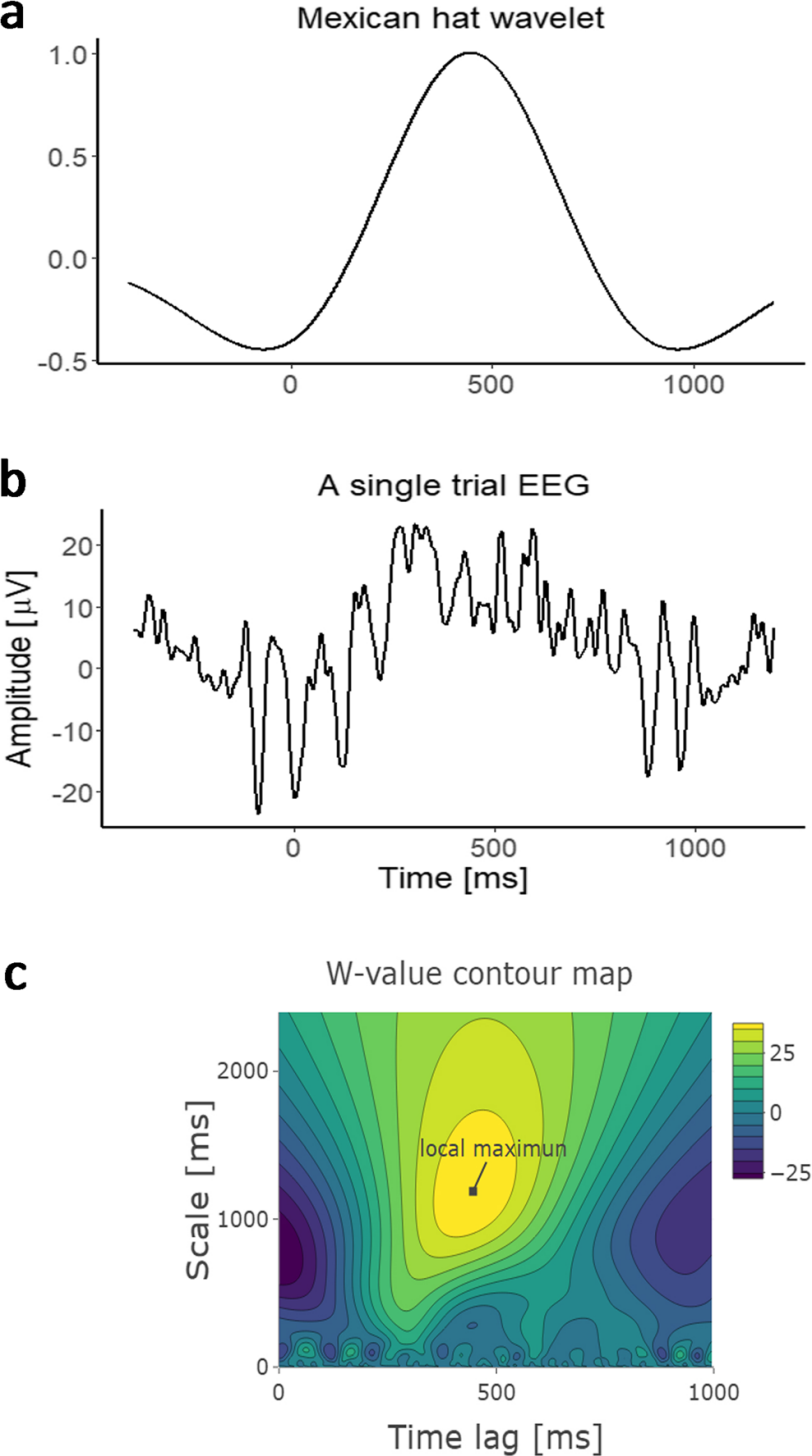
**a** Mexican-hat wavelet (*t* = 446, *s* = 1,188), using the parameters detected as the local extreme in **c**. **b** An example of EEG epoch time-locked to stimulus onset. **c** The resulting *W*-value matrix shown in a contour map when doing the template matching using the trial in **b**. The local extreme detected in the time window of 250 ms ~ 600 ms indicates the single-trial P3. (Color figure online)

Like a template-matching process, the computation involves two arguments: the time lag *t* indicates the peak position of the computed ERP waveform; the scale *s* indicates the breadth of the computed waveform along the *x*-axis (approximately the wavelength; see Fig. 2a). By using a set of *s* and *r* values, the resulting *W*s can be plotted in a contour plot with *t* as the *x*-axis and *s* as the *y*-axis. The local extreme *W* indicates the best matching template of the signal and the *W* at this point gives the measure of the single-trial ERP amplitude (see Fig. 2b–c).

The ERP components of interest are the lateral parietal-occipital P1 and N1, and the parietal P3. We computed P1 as the positive extreme between 50 ms and 150 ms at A10 and B7 in the Biosemi 128-channel system (approximately PO7 and PO8 in the 10–20 system; Di Russo, Martínez, Sereno, Pitzalis, & Hillyard, 2002) and N1 as the negative extreme between 100 ms and 200 ms at the same channels (Hopf, Vogel, Woodman, Heinze, & Luck, 2002). P3 was measured as the positive local extreme between 250 ms and 600 ms at A19 (approximately Pz in the 10–20 system). Each component in each trial can be described by a set of three values: the amplitude *W*, the time lag *t*, and the scale *s*.

#### Time-frequency analysis

The clean EEG signal at A10, A19, B7, and C21 (approximately PO7, Pz, PO8, and Fz in the 10–20 system) was band-pass filtered and Hilbert transformed to be decomposed into alpha (8.5–12 Hz) and theta (4–8 Hz) bands. Channels were selected based on their central positions in frontal (C21), parietal (A19), and bilateral occipital areas (A10 and B7).

For each band, the filter kernel was constructed by the MATLAB function *firls()*. The ideal filter was “plateau-shaped” in a way that the frequency range in the band were the “highland” (set as 1) and the surrounding frequency were “flat” (set as 0). The transition widths were 20%. The length of each filter kernel was long enough to ensure at least three cycles of the lowest frequency in each band. The constructed filter by *firls()* were checked through computing its sum of squared errors (SSE) compared with the ideal filter to ensure it is below 1. After applying the kernel to the EEG signal, the data were Hilbert-transformed by the MATLAB function *hilbert()* to convert to the analytical signal (each data point is in complex form) so that the further computation based on the power or phase information can be performed (Cohen, 2014, Chapter 14). Hilbert transforms allow for the most accurate computation of the signal’s phase, which is crucial for computing oscillatory synchrony.

After transforming the band-pass filtered data into an analytical signal, power was computed as the square of the absolute value at each time point. Coherence was indicated by the intersite phase clustering (ISPC). ISPC were computed through taking the average of phase angle differences between electrodes over time (Cohen, 2014, Chapter 26):

3 $$ ISP{C}_f=\mid {n}^{-1}\sum \limits_{t=1}^n{e}^{i\left({\phi}_{xt}-{\phi}_{yt}\right)}\mid, $$

in which *n* is the number of time points. *ϕ*_*x*_and *ϕ*_*y*_ are phase angles from electrode *x* and *y* at frequency *f*.

Both power and ISPC at each time point were averaged in two periods: baseline (−400–0 ms) and after stimulus onset (0–600 ms) separately.

#### Machine learning

We included the measures of single-trial P1, N1, and P3 as well as power and ISPC at the selected channels in alpha and theta bands as markers for our classifier. In total, we had 25 potential predictors (see Fig. 5) for the classification of mental states in each trial. The machine-learning algorithm used is the support vector machine (SVM) because of its high performance in EEG classification (Lotte, Congedo, Lecuyer, Lamarche, & Arnaldi, 2007). Moreover, SVM does not assume that the relationship between labels and predictors is linear.^3^ Given that we did not have a specific assumption about the relationship between EEG markers and mind-wandering states, we considered SVM to be more appropriate. Markers were *z*-transformed before entering the classifier. SVM learning was performed using the e1071 package in R. A radial kernel (RBF) was performed to allow for the possibility of a nonlinear separating boundary. The optimal regularization parameter *C* and the RBF parameter *γ* were obtained through grid search.

Considering individual differences in EEG patterns, model fitting was performed on each individual. If the data sample size was imbalanced between classes (e.g*.,* if one had a mind-wandering rate of 70%, then 70% of the data were labeled as mind-wandering and 30% as on-task), we copied the cases from the minority class to make the training sample balanced (random oversampling; Chawla, 2005). Models were validated by leave-one-out cross validation (LOOCV). LOOCV is a validation method that in each loop of training, one case from the whole data sample is left to be tested while the rest of the cases form the training sample. The training loop iterates until all the cases have been tested. Performance was measured as prediction accuracy, sensitivity, and specificity*.* Sensitivity is also called the true positive rate. It is calculated as the proportion of positives that are correctly classified as such (i.e., the percentage of mind-wandering trials that are correctly classified as the mind-wandering state). Specificity is also called the true negative rate. It is the proportion of negatives that are correctly classified as such (i.e., the percentage of on-task trials that are correctly classified as the on-task state). Across-task prediction was performed by training data on one task and testing the obtained model on the data of the other task.

Furthermore, to investigate the respective contributions of the EEG markers, we trained models using each marker separately. In this modeling process, we pooled the normalized data from both tasks before training and tested them using LOOCV.

## Results

### Behavioral results

Regarding the probes, participants reported (1) being entirely concentrated on the ongoing task for 29.76% (*SD* = 17.25) of probes, (2) evaluating aspects of the task for 16.48% (*SD* = 9.95) of probes, (3) thinking about personal matters for 22.35% (*SD* = 17.19) of probes, (4) being distracted by their surroundings for 11.16% (*SD* = 7.81) of probes, (5) being daydreaming for 15.37% (*SD* = 9.86) of probes, and (6) not paying attention for 4.88% (*SD* = 5.62) of probes.

The reported mind-wandering rate during task performance varied strongly across participants (*M* = 0.38, *SD* = 0.15, range: 0.16–0.82). The reported mind-wandering rate was lower in the visual search task than in the SART (0.34 vs. 0.42), *t*(17) = 3.62, *p* = .002, *d* = 0.85).

**Fig. 3:**
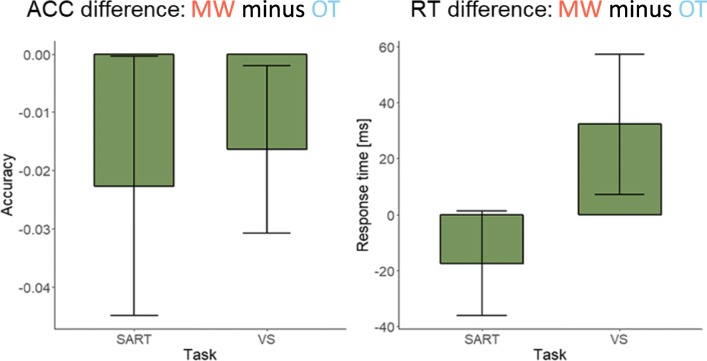
Behavioral results by task. Bars show the behavioral difference (MW minus OT) between conditions. Error bars indicate the 95% confidence interval. ACC = accuracy; RT = response time; MW = mind-wandering; OT = on-task; SART = sustained attention to response task; VS = visual search task

Figure 3 shows the behavioral performance difference between mind-wandering and on-task state (mind-wandering minus on-task). Negative values in the accuracy plot indicate worse performance in mind-wandering than in on-task. Positive values in the response-time plot indicate slower reaction in mind-wandering than in on-task state. The trends in the plots were confirmed by paired *t* tests. Specifically, response accuracy in mind-wandering decreased significantly in the visual search task (0.95 vs. 0.97), *t*(17) = −2.30, *p* = .034, *d* = 0.54, and marginally in the SART (0.93 vs. 0.95), *t*(17) = −2.05, *p* = .056, *d* = 0.48. Response time in mind-wandering increased in the visual search task (687 ms vs. 654 ms) *t*(17) = 2.59, *p* = .019, *d* = 0.61, while in the SART the difference between mind-wandering and on-task was not significant (444 ms vs. 462 ms) *t*(17) = −1.88, *p* = .077, *d* = 0.44.

### Classification results

**Fig. 4:**
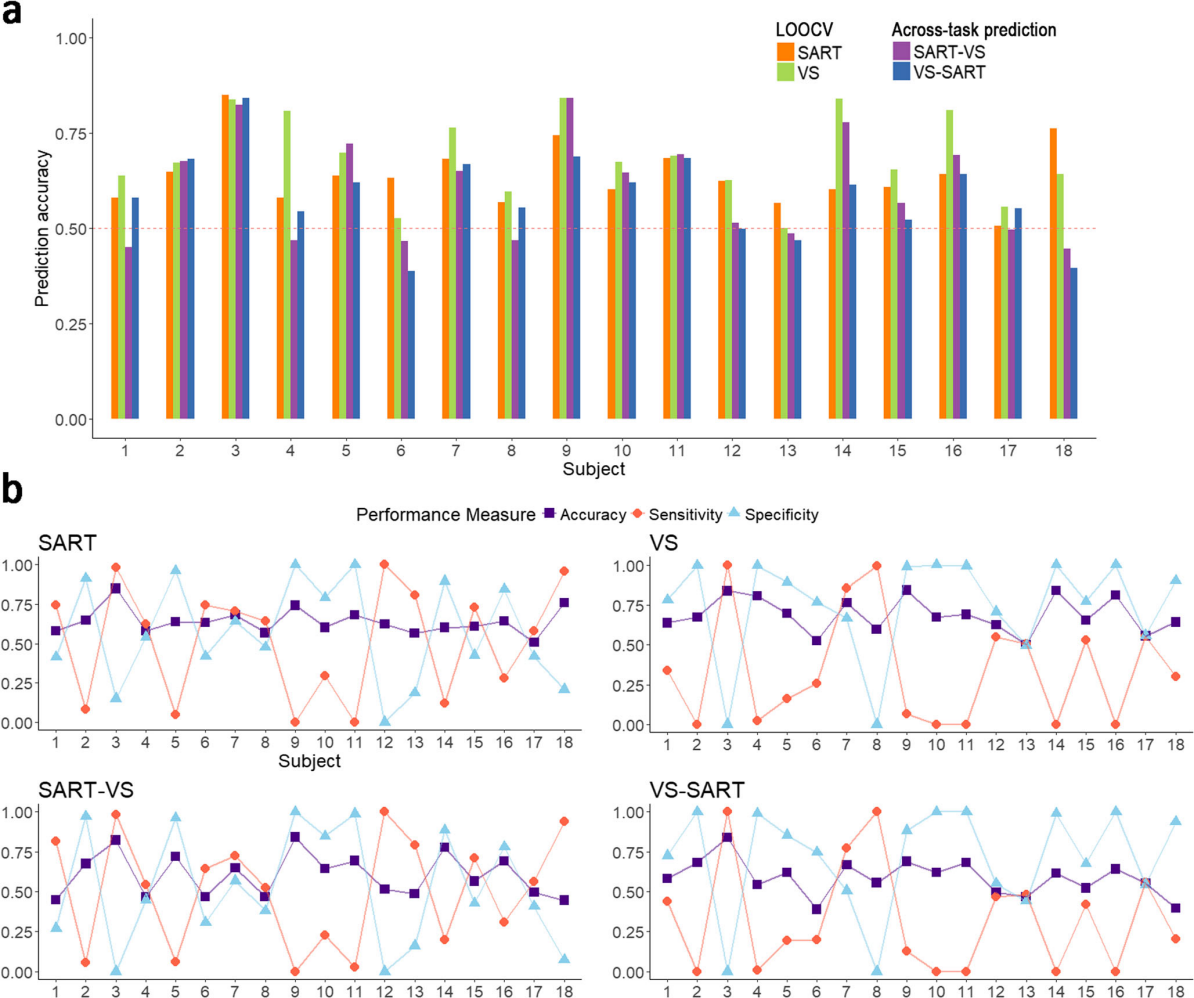
Classifier performance for each participant shown by (**a**) prediction accuracy obtained from the within-task leave-one-out cross validation (LOOCV) and across-task predictions, and (**b**) accuracy, sensitivity, and specificity. Maroon horizontal dashed line in **a** indicates chance level. (Color figure online)

Machine-learning performance for each subject is shown in Fig. 4. For LOOCV, in which training and testing was based on different subsets of the same data set, the prediction accuracy ranged from 0.50 to 0.85 across individual models (*M =* 0.64 for the SART, *M* = 0.69 for the visual search task). For the across-task prediction, we trained models on the basis of the SART data and tested them on data of the visual search task (SART-VS) and vice versa (VS-SART). The obtained prediction accuracy ranged from 0.39 to 0.84 (*M* = 0.60 for SART-VS, *M* = 0.59 for VS-SART). The obtained sensitivity (the percentage of mind-wandering trials that are correctly classified as the mind-wandering state) and specificity (the percentage of on-task trials that are correctly classified as the on-task state) varied considerably across individuals. Sensitivity ranged from zero to one with a mean of 0.42. Specificity ranged from zero to one with a mean of 0.64. Overall, the prediction accuracy is significantly above the chance level of 0.5. A *t* test conducted between the obtained accuracy and 0.5 confirmed this difference in the LOOCV results: *t*(17) = 7.26, *p* < .001, *d* = 1.71 in the SART, and *t*(17) = 7.30, *p* < .001, *d* = 1.72 in the visual search task, as well as in the across-task prediction: *t*(17) = 3.26, *p* = .005, *d* = 0.77 in SART-VS, and *t*(17) = 3.29, *p* = .004, *d* = 0.78 in VS-SART.

**Fig. 5:**
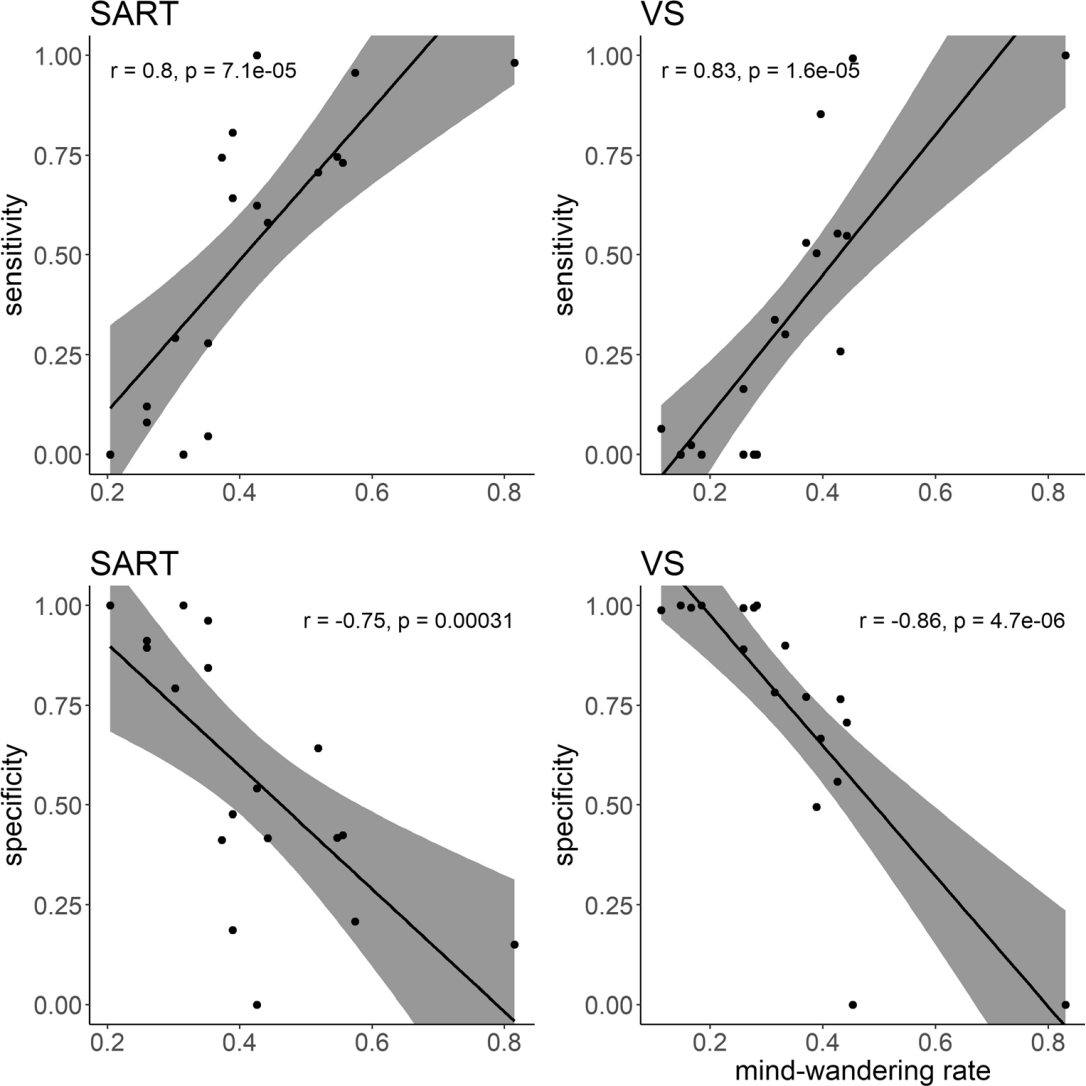
Correlation between mind-wandering rate, sensitivity, and specificity. Shaded area indicates the 95% confidence interval. SART = sustained attention to response task; VS = visual search task. A mind-wandering rate of 1 indicates the participant is mind-wandering every time a probe is presented, whereas a mind-wandering rate of 0 indicates the participant is never mind-wandering when the probe is presented

Through visually inspecting the Fig. 4b, we found the classifier had a bias. In order to find out the possible cause, we did a supplementary Spearman’s rank correlation analysis between mind-wandering rate, sensitivity, and specificity. This analysis showed that sensitivity was positively correlated with the mind-wandering rate during both tasks, *r*(16) = .80, *p* < .001 in the SART; *r*(16) = .83, *p* < .001 in the visual search task, while the specificity was negatively correlated with the mind-wandering rate, *r*(16) = −.75, *p* < .001 in the SART; *r*(16) = −.86, *p* < .001 in the visual search task (see Fig. 5).

### Contributions of individual markers

To find the most predictive EEG marker, we fit models for each EEG marker separately on the full data set including both tasks and performed cross-validation to test the resulting models. Similar to the how we did the whole model fitting process, we found the best parameters (*C* and *γ*) of each single-EEG-marker model by means of a grid search.

**Fig. 6:**
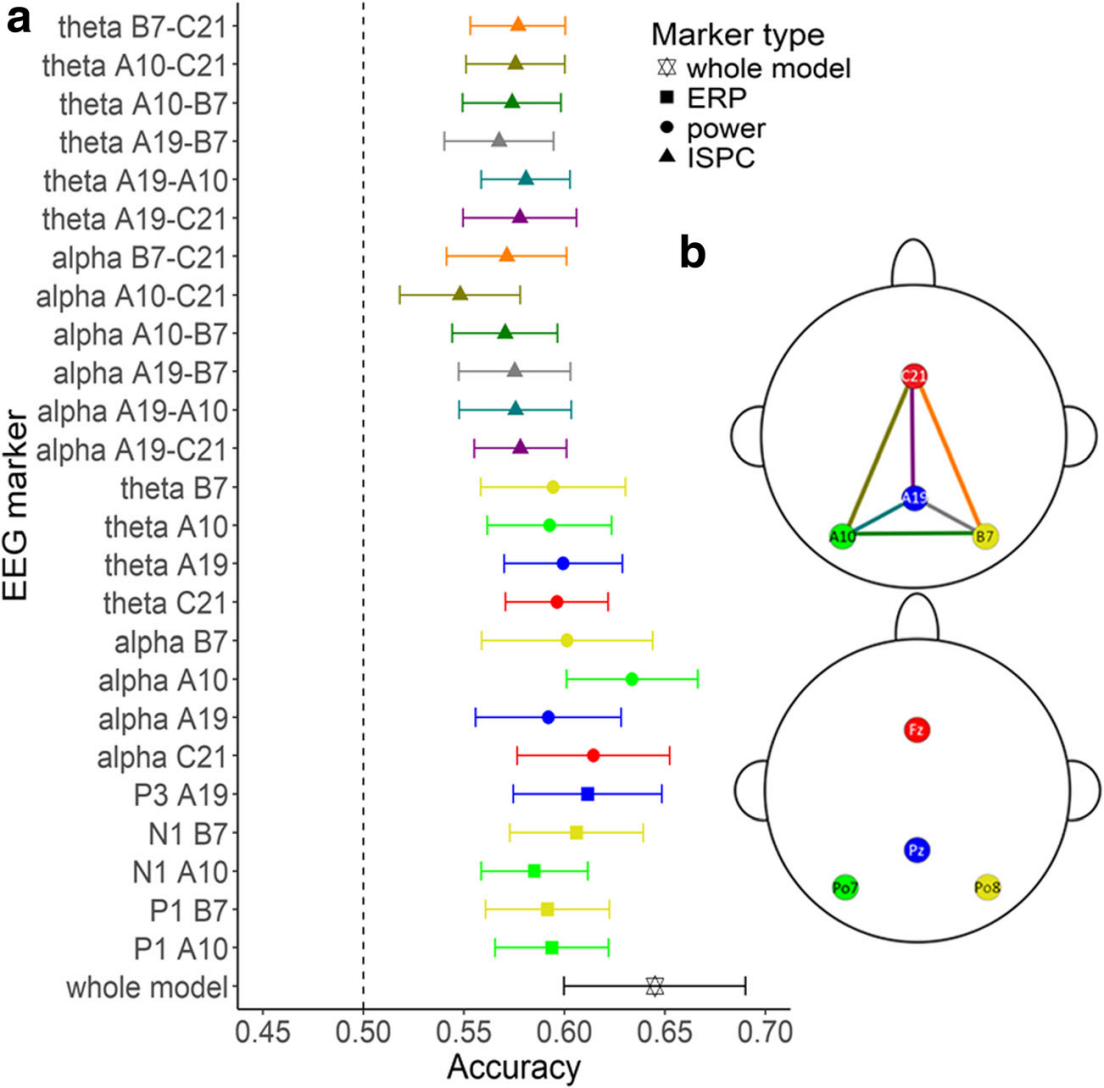
**a** Performance of single-marker classifiers shown as mean accuracy across individuals. Whole model at the bottom refers to the modeling performance with all the EEG markers listed above as predictors. Error bars indicate 95% confidence interval. Black vertical dashed line indicates the chance level. **b** Selected channels to examine in the 128-channel Biosemi system in the upper panel and their approximate locations in the 10–20 system in the lower panel. (Color figure online)

Overall, the performance of each single-marker model was above chance level, which was confirmed by *t* tests (*t*s > 3.24, *p*s < .005; see Fig. 6). The accuracy of the full model including all EEG markers was 0.64 (*SD* = 0.09) on average. Most individual EEG marker models did not reach the performance of the whole model (*t*s < −2.34, *p*s < .032) except for the frontal alpha power (alpha C21): *t*(17) = −0.87, *p* = .393, and the left occipital alpha power (alpha A10): *t*(17) = −1.68, *p* = .11. The equivalent performance between the frontal or left occipital alpha power alone and the whole model was further confirmed by tests of equivalence (Robinson & Froese, 2004). In the equivalence test between alpha C21 and the whole model, the mean difference was −0.03 and the 95% confidence interval of the two one-sided *t* test (TOST) was −0.06 to 0. In the equivalence test between alpha A10 and the whole model, the mean difference was −0.01 and the 95% TOST interval was −0.03 to 0.01. In both cases, the null hypothesis of statistical difference was rejected.

**Fig. 7:**
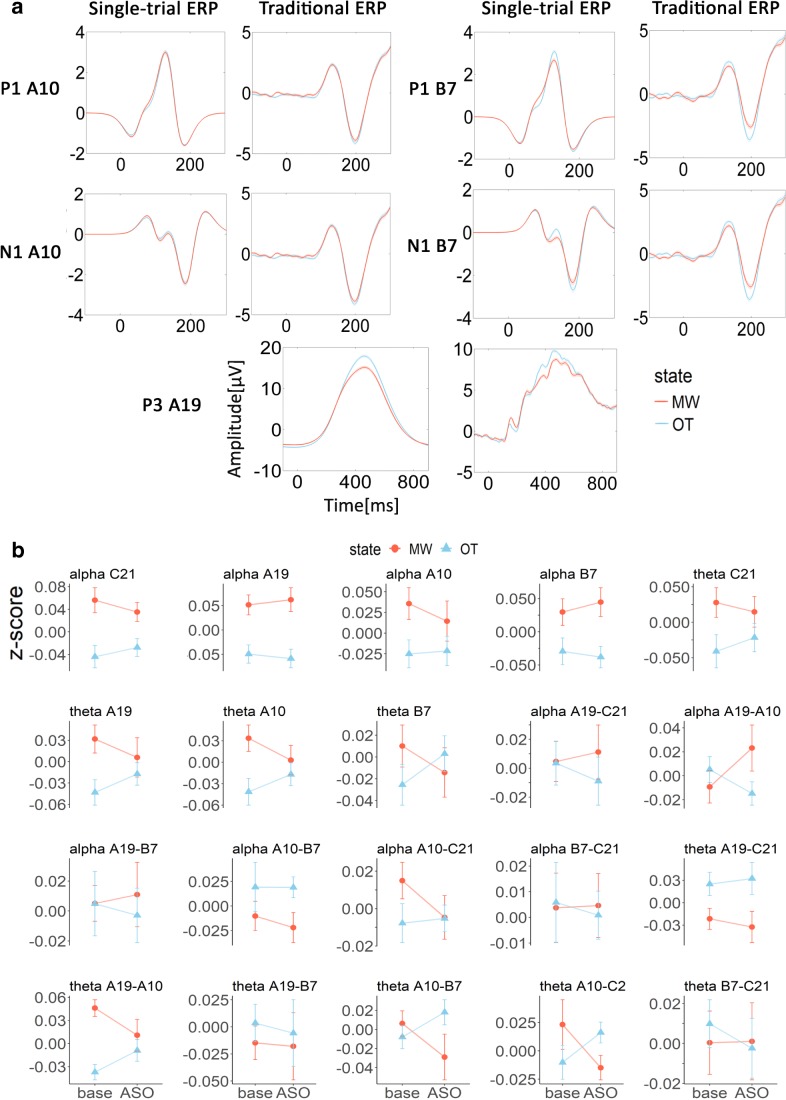
Visualization of the EEG markers in the mind-wandering (MW) and on-task (OT) state. **a** Group averaged ERP wave graph computed by both the single-trial algorithm and the traditional averaging method. Shaded area in the waveform shows the standard error. **b** Group mean of the normalized power and intersite-phase clustering (ISPC) of baseline (−400 ms ~ 0 ms) and after-stimulus onset (ASO, 0 ms ~ 600 ms). Error bar indicates one standard error of the mean. (Color figure online)

To see how these markers differed between the two conditions, we plotted the ERP wave graphs computed by both the traditional averaging method and the single-trial algorithm for mind-wandering and on-task states separately. The average levels of power and ISPC during baseline and after-stimulus onset for mind-wandering and on-task are also shown in Fig. 7. In the ERP markers, a statistically significant difference between on-task and mind-wandering was found with both traditional P3 and single-trial P3 (see Table 1). A difference between on-task and mind-wandering in right occipital P1 (P1 B7) was only found with the single-trial analysis but not with the traditional ERP averaging method. Conversely, the difference between on-task and mind-wandering on the right occipital N1 (N1 B7) was only found with the traditional ERP analysis but not with the single-trial ERP. Note that the peak of N1 obtained through single-trial ERP was slightly earlier (around 180 ms) than the true center of the N1 (around 200 ms). However, for the P1 and the P3, both the single-trial and the traditional ERP centered at almost the same time position. Possible causes for any of the discrepancies are discussed later.

**Table 1:** *T*-test statistics for the comparisons of ERP markers between on-task and mind-wandering, for ERPs computed by both the single-trial algorithm and the traditional averaging method

Single-trial ERP				
Component	Electrode	*t*	*p*	*d*
P1 (120 ms ~ 130 ms)	A10	*ns*		
	B7	2.2	.041	0.36
N1 (175 ms ~ 185 ms)	A10	*ns*		
	B7	*ns*		
P3 (400 ms ~ 500 ms)	A19	3.97	<.001	0.59
Traditional ERP				
Component	Electrode	*t*	*p*	*d*
P1 (120 ms ~ 130 ms)	A10	*ns*		
	B7	*ns*		
N1 (195 ms ~ 205 ms)	A10	*ns*		
	B7	2.78	.012	0.31
P3 (400 ms ~ 500 ms)	A19	3.56	.002	0.50

The time window indicates the interval on the basis of which the mean amplitude was computed. *ns* = nonsignificant

Toward each of the band frequency markers, we performed a two-way repeated ANOVA with state (MW vs. OT) and time (baseline vs. after-stimulus onset) as the within-subjects factors. A statistically significant difference between on-task and mind-wandering was found in frontal alpha power (alpha C21), *F*(1, 17) = 6.37, *p* = .021, η_g_^2^ = 0.23; parietal alpha power (alpha A19), *F*(1, 17) = 9.72, *p* = .006, η_g_^2^ = 0.30; and the coherence between the parietal and the left occipital sites in the theta band (theta A19–A10), *F*(1, 17) = 7.55, *p* = .014, η_g_^2^ = 0.16. Neither a main effect of time (baseline vs. after-stimulus onset), nor any interaction were found.

## Discussion

Our study aimed to find task-independent electrophysiological markers that can differentiate mind-wandering from the on-task state. To achieve this goal, we had participants perform both an inhibition control task that is frequently used to study mind-wandering—the SART—and a visual search task. As in previous studies, we found that mind-wandering disrupted task performance in general. In the SART, this disruption manifested in a trend of decreased accuracy when the participant was mind-wandering. In the visual search task, both accuracy and response time indicated worse performance during the mind-wandering state.

Having established that mind wandering occurred in both tasks and caused disruptions in task performance, we then attempted to predict mind-wandering based on EEG markers. On average, classification accuracy was above chance level. Moreover, even though classification accuracy was not very high, it was task-general: it was possible to train a classifier on one task and use the obtained model to predict the mind-wandering state on another task. Results of our research confirm the potential to use EEG-based machine-learning classifiers to detect mind-wandering, without having to first train on the new tasks. In that way, the detection of mind-wandering could be less interfering and interruptive, allowing us to understand better when, how, and why mind-wandering occurs.

However, several cautionary remarks should be made. Although we were able to classify mind-wandering across tasks, general classification accuracy was still relatively low. This is probably due to the difficult distinction we are trying to make, compared with other EEG classification studies. Whereas most studies focus on classifying different experimental conditions (e.g., Borst, Schneider, Walsh, & Anderson, 2013), in this study we try to classify two different mental states within a single task. Although there were small behavioral differences between the mind-wandering and on-task states, our participants did not stop performing their primary task while mind-wandering, which made the two states highly similar. Unfortunately, this means that the current results cannot be directly used in clinical or industrial applications. If it were to be applied in some industrial application or medical practice like neurofeedback, performing spatial filtering on EEG might be helpful (Blankertz, Tomioka, Lemm, Kawanabe, & Muller, 2008) because it extracts more discriminable EEG markers, which might improve the prediction accuracy. However, spatially filtered EEG markers are not suitable for neurophysiological interpretations because they are computed with the aim of achieving maximum difference between conditions and therefore do not allow us to draw any conclusions about the relative contributions of different EEG markers to mind-wandering.

Second, the unbalanced sensitivity and specificity showed that the models were biased toward detecting one of the two classes (see Fig. 4b). In some individual models, the classifier was good at detecting mind-wandering cases but poor at detecting on-task cases (high sensitivity and low specificity). In some other individual models the classifier was good at detecting on-task cases but poor at detecting mind-wandering cases (low sensitivity and high specificity). In a correlation analysis, we found that individual differences in bias were strongly associated with the amount of mind-wandering during task performance (see Fig. 5). In other words, the more frequently they were mind-wandering, the more biased the model was toward detecting the mind-wandering cases—the trained models were better at detecting the majority class. However, since we balanced the sample size in each class before training the SVM, this cannot be the result of learning the probability of each class. A possibility is that subjects held different standards when they decided their attentional states. Those who engaged more with the primary task might tend to decide their momentary attentional state as on-task. On the contrary, those who engaged more with the mind-wandering process might tend to report off-task thinking. The blurred line between on-task and mind-wandering state when giving self-reports might cause the data to be imprecisely labeled, which further influenced the machine learning result.

In the future, it might be better to have participants rate their attentional levels on a scale rather than to choose between several options. For example, a scale could range from −5 to 5, with −5 indicating the most certain off-task state, 5 indicating the most certain on-task state, and 0 indicating having difficulty to decide. In that way, trials rated 0 could be omitted due to participants’ inability to decide their momentary attentional state. Furthermore, researchers could only analyze those confidently rated cases like the ones with an absolute value above 3, so that it might increase the reliability of the labels.

Our research goes beyond previous studies of how mind-wandering is reflected in EEG activity by using a data-driven method to find what EEG marker is most predictive. While each of the EEG markers individually can be used as a classifier after training, only frontal and the left-occipital alpha power reached a level of performance that was comparable to the complete model. Considering the computational advantage with fewer predictors, this result suggests it may be possible to build a simplified EEG classifier of mind-wandering with only the power in the alpha band at several representative sensors placed at frontal and parietal-occipital sites. However, note that the multifeature SVM did reach the highest accuracy overall (see Fig. 6), and outperformed all logistic regression classifiers (see Fig. 12 in Appendix D). This suggests that classification does not rely on a linear boundary, and that it is not possible to switch to a simple linear classifier.

To understand why these EEG markers can be used to predict mind-wandering, we compared their measures between the mind-wandering and the on-task classes (see Fig. 7). As mentioned above, it should be noted that a straightforward “cutoff” is unlikely to fully explain the relationship that the SVM found. Considering the possibility that SVM can find a nonlinear separating boundary, the state effect that we depicted in Fig. 7 (e.g., alpha A19: mind-wandering > on-task) might only be a likelihood that the SVM used as part of a more complex pattern. This also explains why the SVM can still build classifiers upon those EEG markers that did not show significant mental state differences (e.g., alpha ISPC B7–C21). SVM might have found a nonlinear boundary in the data space formed by such markers.

The set of EEG markers that significantly predicted mind-wandering also informs psychological theories about the mechanisms underlying mind-wandering. For example, the relative reduction in P1 and N1—indices of early sensory processing—support the idea that mind-wandering state is associated with inhibition of sensory processes (“perceptual decoupling”). Besides, the relative reduction of P3, as an indication of the devoted mental efforts to the primary task, in the mind-wandering state is compatible with the cognitive decoupling hypothesis.

Here, the single-trial ERP algorithm showed the advantage in building an efficient classifier through “picking” out the signal from noise within each EEG epoch. The traditional P1 failed to show the subtle difference between on-task and mind-wandering, which was similar to the finding in a recent study by Gonçalves et al. (2018). However, the single-trial P1 magnified this difference and was able to show an effect. An exception to the advantage of the single trial ERP was the seeming absence of a difference between on-task and mind-wandering in the single-trial N1. This lack of an effect is probably related to the poor choice of the time window that we set for N1. After mapping the cross-covariance of the EEG signal and the wavelets into the contour map, we looked for N1 as the local minimum in the time window of 100–200 ms, which resulted in N1s that were all centered before 200 ms. However, judging by the traditional N1 graph, the real N1 peak should be around 200 ms, which means the upper limit of the time window we used to look for N1 might have been too small. A better window would be 100–230 ms. This also explains the earlier peak position of the single-trial N1 in Fig. 7. Thus, the “unsatisfactory performance” of single-trial N1 cannot refute the promising application of this method in analyzing EEG data at the single trial level.

Besides the ERP markers, alpha power at both parietal and frontal sites showed statistically significant differences between on-task and mind-wandering, where mind-wandering was associated with enhanced alpha power. Our findings are consistent with previous studies that found that smaller alpha power predicted higher levels of attentiveness (Macdonald, Mathan, & Yeung, 2011) and were associated with an active attentional suppression mechanism (Kelly, Lalor, Reilly, & Foxe, 2006).

A surprising finding was that theta coherence between the parietal and left occipital sites (theta A19–A10) increased with mind-wandering. In contrast, previous findings associated theta coherence with being more on-task, making it difficult to explain our effect. It is possible that the neural communication between the parietal and occipital cortical area in the theta band might undertake certain complementary functions when sensory inhibition is ongoing in the mind-wandering state so that the task can still be performed. However, further evidence is required to validate this theory.

Another potential future direction in studying EEG markers of mind-wandering could be to investigate the relationship between the separate markers that we identified and broadband EEG power. According to research by Miller, Honey, Hermes, Rao, and Ojemann (2014), power spectral changes can be divided into rhythmic and nonrhythmic EEG. The nonrhythmic part, which is also called the broadband spectral change, is hypothesized to reliably track task engagement. Given that we found significant relationships between EEG features in several different frequency bands, broadband EEG may be a suitable addition to the biomarker of mind-wandering.

To sum up, our research demonstrates the potential for predicting mind-wandering using interpretable electrophysiological markers combined with machine learning. The classifier we developed is task-independent, as we achieved prediction accuracy above chance level in across-task predictions. While each of the EEG markers alone can already detect mind-wandering, we found that alpha power performed equivalently to the whole model and is therefore the most suitable candidate for building a simplified EEG classifier of mind-wandering. This research also supports the idea that mind-wandering is associated with sensory and cognitive decoupling. If our results can be replicated in larger samples, they could potentially be used to detect mind-wandering in real-life situations.

## Electronic supplementary material

ESM 1 (PDF 15.7 mb)

## Footnotes

1 We used six trials preceding each probe to have a large enough sample size. The assumption underlying this method is that the 30 seconds before each probe reflected the same mental state. Considering the possibility that the mind-wandering and on-task states have a shorter duration, we also performed the same analysis on the data of three trials before each probe, accounting for roughly 15–18 seconds. The results are shown in Appendix B: The classifier trained on three preceding trials did not outperform the classifier trained on the six preceding trials.

2 This classification is not the only possibility. For instance, it has been argued that the second option indicates mind-wandering, because “evaluating aspects of the task” could interfere with primary task performance, just like mind-wandering. However, our primary goal is to build a task-general EEG marker of mind-wandering. Mental activity related to task evaluation might be disruptive in simple tasks, but it could be helpful in more complex tasks that involve strategic planning or adjusting behavior based on feedback. Given this reason, we classified Option 2—“I evaluated aspects of the task”—as an on-task state. That said, we also included results based on the other possible categorization, with Answer 1 as on-task and Answer 2, 3, and 5 as mind-wandering in Appendix C.

3 We have also tried a linear classifier—logistic regression—which is supposed to find some simpler “cutoff” rule that can classify mind-wandering versus on-task state in a way like “higher P3 = on-task” and “lower P3 = mind-wandering.” The results can be found in Appendix D. To summarize, logistic regression models performed worse than SVM generally. The results support the need to use the nonlinear power of the SVM.
