# Supplementary material for: Predicting task-general mind-wandering with EEG
Source: Cogn Affect Behav Neurosci. 2019 Mar 8;19(4):1059–73. doi: 10.3758/s13415-019-00707-1 (PMC6711882; doi:10.3758/s13415-019-00707-1)
Supplement: Supplementary file 1 — (PDF 15.7 mb) [file 13415_2019_707_MOESM1_ESM.pdf]

Appendix

A. Word list used in the sustained-attention-to-response task (SART)

pleasant	number	backward	breath	address	lawyer	agreed	all
America	American	fear	poor	band	bank	bar	thanks
shall	aim	business	bone	burial	pretty	both	important
belt	prepared	message	judgment	bed	discuss	existence	pay
mean	better	respect	clouded	turn	like	command	move
property	worried	special	shortly	blue	book	bunch	message
boat	break	write	break	desk	believed	roof	close
thick	love	continue	run	running	continue	courage	eternal
English	single	huge	enormous	behind	experience	party	fact
guest	guest	hole	soon	area	left	building	fuss
eat	help	lied	sound	commonly	pleasure	called	caught
talk	history	written	closed	crazy	stop	prison	feelings
conscience	injured	normal	sit	family	search	glass	evening
throw	joke	half	lord	hero	real	angle	high
hundred	hope	hotel	marriage	ice	impression	suddenly	card
knowledge	kitchen	sale	short	cost	newspaper	kiss	pillow
smile	burden	live	age	leather	read	army	leading
class	alive	lying	lies	lift	list	left	lot
lazy	listening	succeed	size	most	girls	middle	minute
mist	beautiful	wall	think	thought	nine	neck	above
call	morning	explore	incredible	immediately	breakfast	discovered	receive
view	uncle	get	hurry	ears	old	seem	survival
dad	partner	adjust	suit	place	position	beautiful	hit
weird	right	accounting	account	relationship	running	drive	risk
red	call	grey	smoke	quarrel	appears	shoes	clean
bolt	write	second	simple	sleep	battle	keys	hit
lock	understand	some	special	jump	stuff	insert	steal
chair	stop	slice	briefcase	restraint	drawing	right	back
much	happy	time	consent	coincidence	total	stairs	pull
faith	twelve	twenty	hours	holiday	often	morning	security
many	change	responsibility	verb	disappear	forgive	declaration	solid
loss	difference	fresh	chill	departure	left	celebrate	enemy
right	meat	airplane	flight	foot	feet	follow	complete
adult	peace	boyfriend	where	to	when	which	income
weekend	desire	under	residential	see	sea	looking	sun
will	heavy	black					

B. Modelling performance based on three preceding trials from each probe

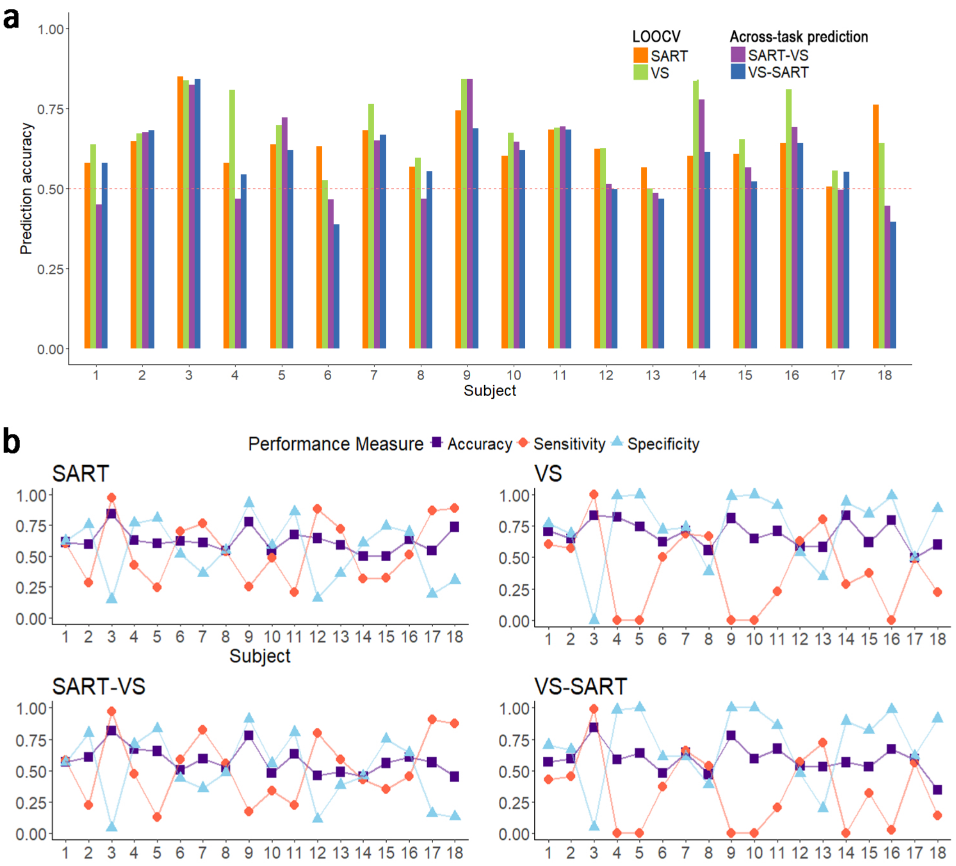

Figure I. Modelling performance for both within-task leave-one-out cross-validation (LOOCV) and across-task prediction based on 3 preceding trials of each probe. The mean accuracy for the LOOCV was 0.62 (*SD* = 0.09) in the SART and 0.69 (*SD* = 0.10) in the visual search task (VS). For the across-task predictions, the mean accuracy was 0.58 (*SD* = 0.11) for testing the SART model on the data of the visual search task (SART-VS) and 0.59 (*SD* = 0.11) for testing the visual search task model on data of the SART (VS-SART). A *t*-test conducted between the obtained accuracy and 0.5 confirmed this difference in the LOOCV: $t(17) = 5.73$, $p < 0.001$, $d = 1.35$in the SART and $t(17) = 7.50$, $p < 0.001$, $d = 1.77$ in the visual search task, as well as in the across-task prediction: $t(17) = 3.21$, $p = 0.005$, $d = 0.76$ in SART-VS and $t(17) = 3.46$, $p = 0.003$, $d = 0.81$ in VS-SART.

C. Mind-wandering classification based on considering evaluation of task performance part of mind-wandering

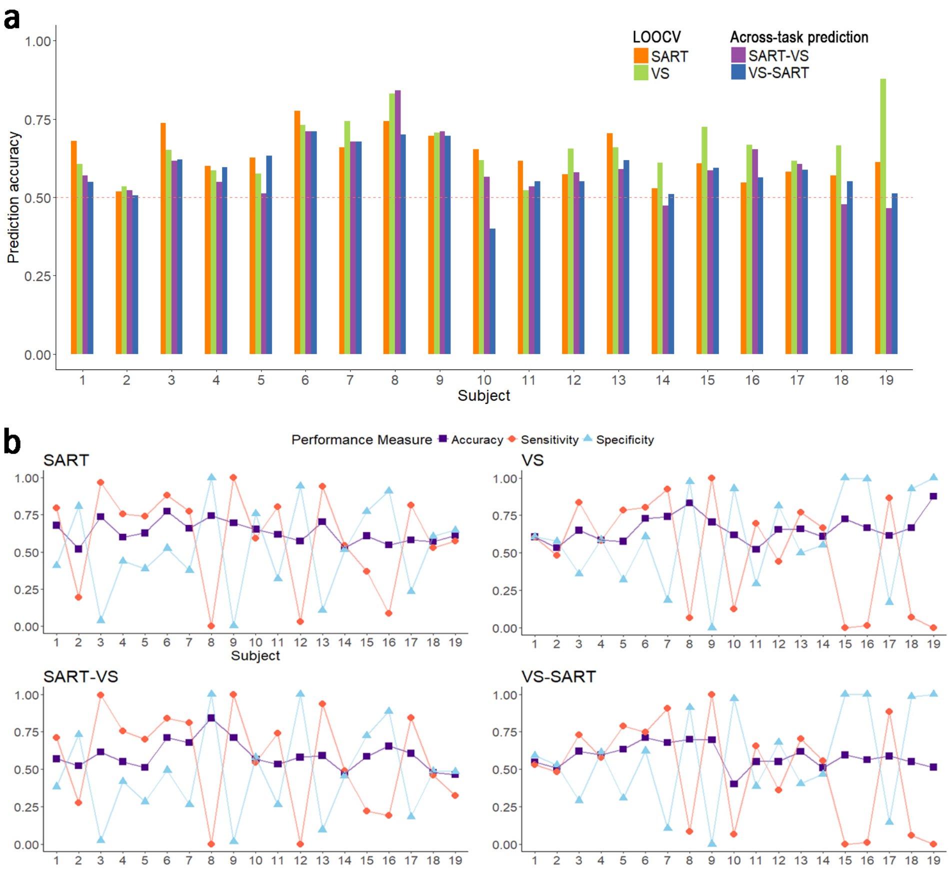

Figure II. Modelling performance based on the categorization that the on-task state referred to answer 1 and the mind-wandering state referred to answer 2, 3, and 5. Note based on this categorization, 19 participants were included. The mean accuracy for the LOOCV was 0.63 (*SD* = 0.07) in the SART and 0.66 (*SD* = 0.09) in the visual search task (VS). For the across-task predictions, the mean accuracy was 0.59 (*SD* = 0.09) for testing the SART model on the visual search task data (SART-VS) and 0.59 (*SD* = 0.07) for testing the visual search task model on data of the SART (VS-SART). A *t*-test conducted comparing the obtained accuracy to the chance level of 0.5 confirmed a significant difference in both within-task LOOCV: $t(18) = 7.81$, $p < 0.001$, $d = 1.79$ in the SART and $t(18) = 7.70$, $p < 0.001$, $d = 1.77$ in the visual search task, as well as in the across-task prediction: $t(18) = 4.19$, $p < 0.001$, $d = 0.96$ in SARTVS and $t(18) = 4.74$, $p < 0.001$, $d = 1.09$ in VS-SART.

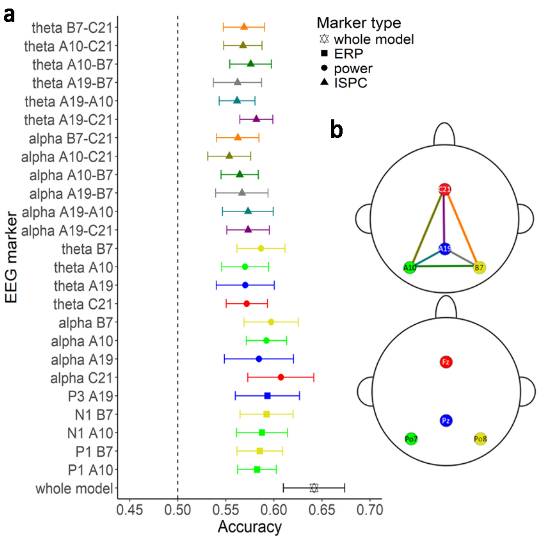

Figure III. Marker testing results based on the categorization that the on-task state referred to answer 1 and the mind-wandering state referred to answer 2, 3, and 5. Paired t-tests showed all the marker alone could predict above the chance level (*ts* > 4.68, *ps* < 0.001). None of the markers outperformed the whole model (*ts* < –2.96, *ps* < 0.008). Error bars indicate 95 percent confidence interval.

D. Performance of logistic regression classifiers

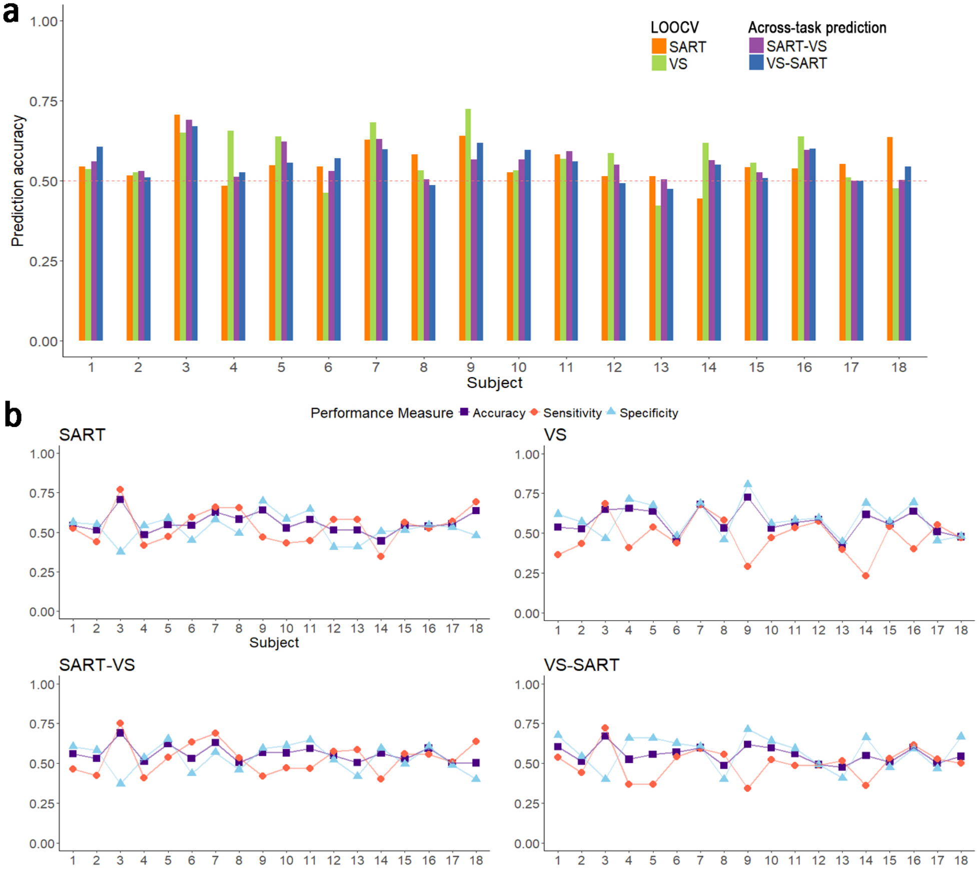

Figure IV. Modelling performance of logistic regression classifiers. The mean accuracy for within-task LOOCV was 0.56 ($SD = 0.06$) in the SART and 0.57 ($SD = 0.08$) in the visual search task (VS). For the across-task predictions, the mean accuracy was 0.56 ($SD = 0.05$) for testing the SART model on the visual search task data (SART-VS) and 0.55 ($SD = 0.05$) for testing the visual search task model on data of the SART (VS-SART). A *t*-test comparing the obtained accuracy to the chance level of 0.5 confirmed this difference in the LOOCV: $t(17) = 3.89$, $p = 0.001$, $d = 0.92$ in the SART and $t(17) = 3.78$, $p = 0.002$, $d = 0.89$ in the visual search task, as well as in the across-task prediction: $t(17) = 4.70$, $p < 0.001$, $d = 1.11$ in SART-VS and $t(17) = 4.20$, $p < 0.001$, $d = 0.99$ in VS-SART. Paired *t*-test results showed the accuracy of logistic regression models was lower than the SVM models in the cross-validation process ($ts > 6.70$, $ps < 0.001$), but no statistically significant difference was found in the acrosstask predictions.

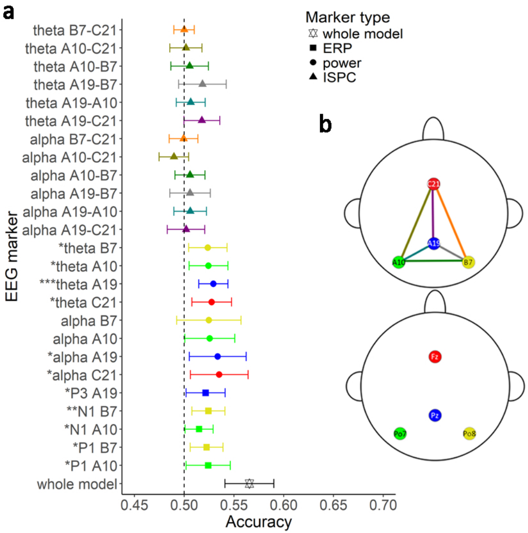

Figure V. Marker testing results using logistic regression as classifiers. Paired *t*-tests showed only 11 markers out of 30 performed above chance level as marked in the graph (*$p < 0.05$, **$p < 0.01$, ***$p < 0.001$). Paired *t*-tests showed the performance of single-marker models built using logistic regression were generally worse than built using SVM (*ts* > 3.37, *ps* < 0.004). Error bars indicate 95 percent confidence interval.
